# Supplementary material for: AffyMAPSDetector: a software tool to characterize Affymetrix GeneChip™ expression arrays with respect to SNPs
Source: BMC Bioinformatics. 2007 Jul 30;8:276. doi: 10.1186/1471-2105-8-276 (PMC1959249; doi:10.1186/1471-2105-8-276)
Supplement: Additional file 13 — AffyMAPSDetector v1 source code. AffyMAPSDetector source code. [file 1471-2105-8-276-S13.zip › AffyMAPSDetector_v1_src/doc/affychip/snpdetector/model/DataState.html]

DataState


|  |  |  |  |
| --- | --- | --- | --- |
| **Overview** \| **Package** \| **Class** \| **Use** \| **Tree** \| **Deprecated** \| **Index** \| **Help** | | |  |
| PREV CLASS   **NEXT CLASS** | **FRAMES**    **NO FRAMES**     **All Classes** |  |  |
| SUMMARY: NESTED \| FIELD \| CONSTR \| METHOD | DETAIL: FIELD \| CONSTR \| METHOD |  |  |


---


## affychip.snpdetector.model Class DataState

```
java.lang.Object
  affychip.snpdetector.model.DataState
```

**All Implemented Interfaces:**: java.io.Serializable

---

public class **DataState** extends java.lang.Object implements java.io.Serializable

Title: Probe SNP Detector

Description: Respository to maintain AffyMapsDetector data state. For
example: gene information file, probeset information file, key fields in both
the files, output data files that store the information collected from web by
AffyMapsDetector etc./p>

Copyright: Sunita Kumari -- Copyright (c) 2004

Company: George Mason University

**Version:**
:   1.1

**Author:**
:   Sunita Kumari

**See Also:**: Serialized Form

---

|  |  |
| --- | --- |
| **Method Summary** | |
| `void` | `clearState()` |
| `int` | `getGeneIdentifierFieldIndex()` |
| `GeneInfo[]` | `getGeneInfo()` |
| `java.lang.String` | `getGeneInfoFieldSeparator()` |
| `java.io.File` | `getGeneInfoFile()` |
| `java.lang.String[]` | `getGeneInfoHeaders()` |
| `java.util.HashMap` | `getGeneInfoMap()` |
| `int` | `getGeneInfoProbeSetIdFieldIndex()` |
| `java.lang.String` | `getHasSnpsFilename()` |
| `static DataState` | `getInstance()` |
| `int` | `getLocusLinkFieldIndex()` |
| `int` | `getMmPosFieldIndex()` |
| `java.lang.String` | `getNoLocusLinkFilename()` |
| `java.lang.String` | `getNoSnpsFilename()` |
| `java.lang.String` | `getOrganism()` |
| `java.lang.String` | `getProbeInfoFieldSeparator()` |
| `java.io.File` | `getProbeInfoFile()` |
| `java.lang.String[]` | `getProbeInfoHeaders()` |
| `java.util.HashMap` | `getProbeInfoMap()` |
| `int` | `getProbeInfoProbeSetIdFieldIndex()` |
| `int` | `getProbeSeqFieldIndex()` |
| `java.util.HashMap` | `getProbeSet()` |
| `java.lang.String` | `getWebGeneInfoFilename()` |
| `java.lang.String` | `getWebSnpInfoFilename()` |
| `int` | `getXPosFieldIndex()` |
| `int` | `getYPosFieldIndex()` |
| `void` | `setGeneIdentifierFieldIndex(int geneIdentifierFieldIndex)` |
| `void` | `setGeneIdentifierFieldIndex(java.lang.Integer geneIdentifierFieldIndex)` |
| `void` | `setGeneIdentifierFieldIndex(java.lang.String geneIdentifierFieldIndex)` |
| `void` | `setGeneInfo(GeneInfo[] geneInfo)` |
| `void` | `setGeneInfoFieldSeparator(java.lang.String geneInfoFieldSeparator)` |
| `void` | `setGeneInfoFile(java.io.File geneInfoFile)` |
| `void` | `setGeneInfoHeaders(java.lang.String[] geneInfoHeaders)` |
| `void` | `setGeneInfoProbeSetIdFieldIndex(int geneInfoProbeSetIdFieldIndex)` |
| `void` | `setGeneInfoProbeSetIdFieldIndex(java.lang.Integer geneInfoProbeSetIdFieldIndex)` |
| `void` | `setGeneInfoProbeSetIdFieldIndex(java.lang.String geneInfoProbeSetIdFieldIndex)` |
| `void` | `setHasSnpsFilename(java.lang.String hasSnpsFilename)` |
| `void` | `setLocusLinkFieldIndex(int locusLinkFieldIndex)` |
| `void` | `setLocusLinkFieldIndex(java.lang.Integer locusLinkFieldIndex)` |
| `void` | `setLocusLinkFieldIndex(java.lang.String locusLinkFieldIndex)` |
| `void` | `setMmPosFieldIndex(int mmPosFieldIndex)` |
| `void` | `setMmPosFieldIndex(java.lang.Integer mmPosFieldIndex)` |
| `void` | `setMmPosFieldIndex(java.lang.String mmPosFieldIndex)` |
| `void` | `setNoLocusLinkFilename(java.lang.String noLocusLinkFilename)` |
| `void` | `setNoSnpsFilename(java.lang.String noSnpsFilename)` |
| `void` | `setOrganism(java.lang.String orgn)` |
| `void` | `setProbeInfoFieldSeparator(java.lang.String probeInfoFieldSeparator)` |
| `void` | `setProbeInfoFile(java.io.File probeInfoFile)` |
| `void` | `setProbeInfoHeaders(java.lang.String[] probeInfoHeaders)` |
| `void` | `setProbeInfoProbeSetIdFieldIndex(int probeInfoProbeSetIdFieldIndex)` |
| `void` | `setProbeInfoProbeSetIdFieldIndex(java.lang.Integer probeInfoProbeSetIdFieldIndex)` |
| `void` | `setProbeInfoProbeSetIdFieldIndex(java.lang.String probeInfoProbeSetIdFieldIndex)` |
| `void` | `setProbeSeqFieldIndex(int probeSeqFieldIndex)` |
| `void` | `setProbeSeqFieldIndex(java.lang.Integer probeSeqFieldIndex)` |
| `void` | `setProbeSeqFieldIndex(java.lang.String probeSeqFieldIndex)` |
| `void` | `setProbeSet(java.util.HashMap probeSet)` |
| `void` | `setWebGeneInfoFilename(java.lang.String webGeneInfoFilename)` |
| `void` | `setWebSnpInfoFilename(java.lang.String webSnpInfoFilename)` |
| `void` | `setXPosFieldIndex(int xPosFieldIndex)` |
| `void` | `setXPosFieldIndex(java.lang.Integer xPosFieldIndex)` |
| `void` | `setXPosFieldIndex(java.lang.String xPosFieldIndex)` |
| `void` | `setYPosFieldIndex(int yPosFieldIndex)` |
| `void` | `setYPosFieldIndex(java.lang.Integer yPosFieldIndex)` |
| `void` | `setYPosFieldIndex(java.lang.String yPosFieldIndex)` |

**Methods inherited from class java.lang.Object**

`clone, equals, finalize, getClass, hashCode, notify, notifyAll, toString, wait, wait, wait`

**Method Detail**

### getInstance

```
public static DataState getInstance()
```

---


### getGeneInfoMap

```
public java.util.HashMap getGeneInfoMap()
```

---


### getProbeInfoMap

```
public java.util.HashMap getProbeInfoMap()
```

---


### getGeneIdentifierFieldIndex

```
public int getGeneIdentifierFieldIndex()
```

---


### getGeneInfoFieldSeparator

```
public java.lang.String getGeneInfoFieldSeparator()
```

---


### getGeneInfoFile

```
public java.io.File getGeneInfoFile()
```

---


### getGeneInfoHeaders

```
public java.lang.String[] getGeneInfoHeaders()
```

---


### getGeneInfoProbeSetIdFieldIndex

```
public int getGeneInfoProbeSetIdFieldIndex()
```

---


### getHasSnpsFilename

```
public java.lang.String getHasSnpsFilename()
```

---


### getLocusLinkFieldIndex

```
public int getLocusLinkFieldIndex()
```

---


### getMmPosFieldIndex

```
public int getMmPosFieldIndex()
```

---


### getNoLocusLinkFilename

```
public java.lang.String getNoLocusLinkFilename()
```

---


### getNoSnpsFilename

```
public java.lang.String getNoSnpsFilename()
```

---


### getProbeInfoFieldSeparator

```
public java.lang.String getProbeInfoFieldSeparator()
```

---


### getProbeInfoFile

```
public java.io.File getProbeInfoFile()
```

---


### getProbeInfoHeaders

```
public java.lang.String[] getProbeInfoHeaders()
```

---


### getProbeInfoProbeSetIdFieldIndex

```
public int getProbeInfoProbeSetIdFieldIndex()
```

---


### getProbeSeqFieldIndex

```
public int getProbeSeqFieldIndex()
```

---


### getWebGeneInfoFilename

```
public java.lang.String getWebGeneInfoFilename()
```

---


### getWebSnpInfoFilename

```
public java.lang.String getWebSnpInfoFilename()
```

---


### getXPosFieldIndex

```
public int getXPosFieldIndex()
```

---


### getYPosFieldIndex

```
public int getYPosFieldIndex()
```

---


### getGeneInfo

```
public GeneInfo[] getGeneInfo()
```

---


### getProbeSet

```
public java.util.HashMap getProbeSet()
```

---


### setYPosFieldIndex

```
public void setYPosFieldIndex(int yPosFieldIndex)
```

---


### setYPosFieldIndex

```
public void setYPosFieldIndex(java.lang.Integer yPosFieldIndex)
```

---


### setYPosFieldIndex

```
public void setYPosFieldIndex(java.lang.String yPosFieldIndex)
```

---


### setXPosFieldIndex

```
public void setXPosFieldIndex(int xPosFieldIndex)
```

---


### setXPosFieldIndex

```
public void setXPosFieldIndex(java.lang.Integer xPosFieldIndex)
```

---


### setXPosFieldIndex

```
public void setXPosFieldIndex(java.lang.String xPosFieldIndex)
```

---


### setWebSnpInfoFilename

```
public void setWebSnpInfoFilename(java.lang.String webSnpInfoFilename)
```

---


### setWebGeneInfoFilename

```
public void setWebGeneInfoFilename(java.lang.String webGeneInfoFilename)
```

---


### setProbeSeqFieldIndex

```
public void setProbeSeqFieldIndex(int probeSeqFieldIndex)
```

---


### setProbeSeqFieldIndex

```
public void setProbeSeqFieldIndex(java.lang.Integer probeSeqFieldIndex)
```

---


### setProbeSeqFieldIndex

```
public void setProbeSeqFieldIndex(java.lang.String probeSeqFieldIndex)
```

---


### setProbeInfoProbeSetIdFieldIndex

```
public void setProbeInfoProbeSetIdFieldIndex(int probeInfoProbeSetIdFieldIndex)
```

---


### setProbeInfoProbeSetIdFieldIndex

```
public void setProbeInfoProbeSetIdFieldIndex(java.lang.Integer probeInfoProbeSetIdFieldIndex)
```

---


### setProbeInfoProbeSetIdFieldIndex

```
public void setProbeInfoProbeSetIdFieldIndex(java.lang.String probeInfoProbeSetIdFieldIndex)
```

---


### setProbeInfoHeaders

```
public void setProbeInfoHeaders(java.lang.String[] probeInfoHeaders)
```

---


### setProbeInfoFile

```
public void setProbeInfoFile(java.io.File probeInfoFile)
```

---


### setProbeInfoFieldSeparator

```
public void setProbeInfoFieldSeparator(java.lang.String probeInfoFieldSeparator)
```

---


### setNoSnpsFilename

```
public void setNoSnpsFilename(java.lang.String noSnpsFilename)
```

---


### setNoLocusLinkFilename

```
public void setNoLocusLinkFilename(java.lang.String noLocusLinkFilename)
```

---


### setMmPosFieldIndex

```
public void setMmPosFieldIndex(int mmPosFieldIndex)
```

---


### setMmPosFieldIndex

```
public void setMmPosFieldIndex(java.lang.Integer mmPosFieldIndex)
```

---


### setMmPosFieldIndex

```
public void setMmPosFieldIndex(java.lang.String mmPosFieldIndex)
```

---


### setLocusLinkFieldIndex

```
public void setLocusLinkFieldIndex(int locusLinkFieldIndex)
```

---


### setLocusLinkFieldIndex

```
public void setLocusLinkFieldIndex(java.lang.Integer locusLinkFieldIndex)
```

---


### setLocusLinkFieldIndex

```
public void setLocusLinkFieldIndex(java.lang.String locusLinkFieldIndex)
```

---


### setHasSnpsFilename

```
public void setHasSnpsFilename(java.lang.String hasSnpsFilename)
```

---


### setGeneInfoProbeSetIdFieldIndex

```
public void setGeneInfoProbeSetIdFieldIndex(int geneInfoProbeSetIdFieldIndex)
```

---


### setGeneInfoProbeSetIdFieldIndex

```
public void setGeneInfoProbeSetIdFieldIndex(java.lang.Integer geneInfoProbeSetIdFieldIndex)
```

---


### setGeneInfoProbeSetIdFieldIndex

```
public void setGeneInfoProbeSetIdFieldIndex(java.lang.String geneInfoProbeSetIdFieldIndex)
```

---


### setGeneInfoHeaders

```
public void setGeneInfoHeaders(java.lang.String[] geneInfoHeaders)
```

---


### setGeneInfoFile

```
public void setGeneInfoFile(java.io.File geneInfoFile)
```

---


### setGeneInfoFieldSeparator

```
public void setGeneInfoFieldSeparator(java.lang.String geneInfoFieldSeparator)
```

---


### setGeneIdentifierFieldIndex

```
public void setGeneIdentifierFieldIndex(int geneIdentifierFieldIndex)
```

---


### setGeneIdentifierFieldIndex

```
public void setGeneIdentifierFieldIndex(java.lang.Integer geneIdentifierFieldIndex)
```

---


### setGeneIdentifierFieldIndex

```
public void setGeneIdentifierFieldIndex(java.lang.String geneIdentifierFieldIndex)
```

---


### setProbeSet

```
public void setProbeSet(java.util.HashMap probeSet)
```

---


### setGeneInfo

```
public void setGeneInfo(GeneInfo[] geneInfo)
```

---


### clearState

```
public void clearState()
```

---


### setOrganism

```
public void setOrganism(java.lang.String orgn)
```

---


### getOrganism

```
public java.lang.String getOrganism()
```


---


|  |  |  |  |
| --- | --- | --- | --- |
| **Overview** \| **Package** \| **Class** \| **Use** \| **Tree** \| **Deprecated** \| **Index** \| **Help** | | |  |
| PREV CLASS   **NEXT CLASS** | **FRAMES**    **NO FRAMES**     **All Classes** |  |  |
| SUMMARY: NESTED \| FIELD \| CONSTR \| METHOD | DETAIL: FIELD \| CONSTR \| METHOD |  |  |


---
